# Protective Effect of Vaccine Doses and Antibody Titers Against SARS-CoV-2 Infection in Kidney Transplant Recipients

**DOI:** 10.3389/ti.2023.11196

**Published:** 2023-06-13

**Authors:** Chien-Chia Chen, Meng-Kai Hsu, Yi-Jen Huang, Mei-Jun Lai, Shu-Wei Wu, Min-Huey Lin, Hsu-Shan Hung, Yu-Chun Lin, Yu-Tsung Huang, Ya-Fen Lee, Meng-Kun Tsai, Chih-Yuan Lee

**Affiliations:** ^1^ Department of Surgery, National Taiwan University Hospital, Taipei, Taiwan; ^2^ Department of Pharmacy, National Taiwan University Hospital, Taipei, Taiwan; ^3^ Department of Laboratory Medicine, National Taiwan University Hospital, Taipei, Taiwan; ^4^ Department of Nursing, National Taiwan University Hospital, Taipei, Taiwan

**Keywords:** kidney transplant, antibody titer, severe acute respiratory syndrome coronavirus 2, vaccine, interferon-γ

## Abstract

Patients undergoing kidney transplantation have a poor response to vaccination and a higher risk of disease progression of severe acute respiratory syndrome coronavirus 2 (SARS-CoV-2). The effectiveness of vaccine doses and antibody titer tests against the mutant variant in these patients remains unclear. We retrospectively analyzed the risk of SARS-CoV-2 infection in a single medical center according to vaccine doses and immune responses before the outbreak. Among 622 kidney transplant patients, there were 77 patients without vaccination, 26 with one dose, 74 with two doses, 357 with three, and 88 with four doses. The vaccination status and infection rate proportion were similar to the general population. Patients undergoing more than three vaccinations had a lower risk of infection (odds ratio = 0.6527, 95% CI = 0.4324–0.9937) and hospitalization (odds ratio = 0.3161, 95% CI = 0.1311–0.7464). Antibody and cellular responses were measured in 181 patients after vaccination. Anti-spike protein antibody titer of more than 1,689.3 BAU/mL is protective against SARS-CoV-2 infection (odds ratio = 0.4136, 95% CI = 0.1800–0.9043). A cellular response by interferon-γ release assay was not correlated with the disease (odds ratio = 1.001, 95% CI = 0.9995–1.002). In conclusion, despite mutant strain, more than three doses of the first-generation vaccine and high antibody titers provided better protection against the omicron variant for a kidney transplant recipient.

## Graphical Abstract

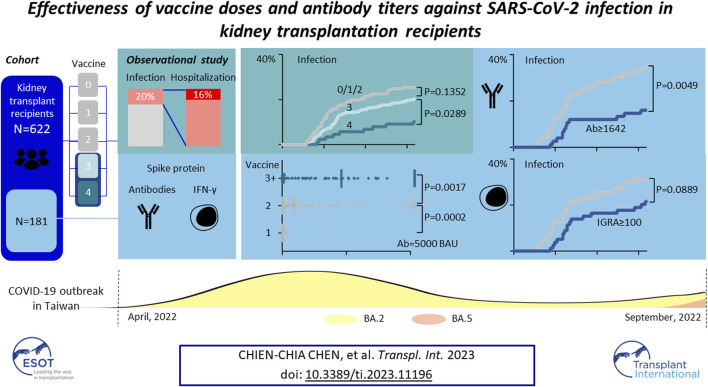

## Introduction

Despite multiple doses, patients have a poor response to vaccines against severe acute respiratory syndrome coronavirus 2 (SARS-CoV-2) after solid organ transplantation [1–4] compared to the response in immunocompetent population. Moreover, the disease severity is greater in this population. Higher hospitalization and more severe complication rates with significant mortality were reported since the coronavirus disease 2019 (COVID-19) pandemic in early 2020 [5, 6].

Since January 2022, the SARS-CoV-2 Omicron variant has overtaken previous variants and dominated the pandemic. Multiple mutations in the spike protein rendered the Omicron variant a higher affinity for angiotensin-converting enzyme 2 receptor and a lower ability to use the serine protease TMPRSS2 [7, 8]. Compared with the delta variant, these changes made the Omicron variant more transmissible but reduced its severity and risk of mortality [9].

Nevertheless, for Solid organ transplant recipients (SOTR) with an immunodeficient status, there was still a higher risk of hospitalization and mortality than in the general population [10, 11]. Although vaccination against SARS-CoV-2 with repeated boosters is recommended for SOTR, there is a concern for the decreased effect of the first generation of COVID-19 vaccines against the Omicron strain [12]. A higher titer of anti-spike protein antibody is needed to achieve the protection [13], which is usually not fulfilled in SOTR. A cohort study in Canada reported improved effectiveness by the third dose in SOTR [14], but was still lesser than in the general population. Hence, is it necessary to receive a fourth dose or more vaccines in SOTR? Measuring the antibody titer for SOTR may help [15], but there is no consensus on this issue yet [16].

In Taiwan, before the late epidemic outbreak of Omicron variant BA.2 from April to August 2022, there were only scanty COVID-19 cases, and majority of the population received multiple doses of vaccination [17]. We conducted this retrospective study in kidney transplant recipients (KTR) to evaluate the effectiveness of the first-generation vaccine against the Omicron variant. Besides, some patients underwent measurement of antibody and cellular response after vaccination. The relationship between infection risk and laboratory results was also explored.

## Materials and Methods

This prospective observational study was approved by the Research Ethics Committee of the National Taiwan University Hospital (NTUH) (202106046RINA).

### Patients

Taiwan, an island country located in the west Pacific Ocean, with a population of about 24 million, which makes the assessments of immigration and infectious disease control easy. Since late January 2022, strict epidemic prevention policies have been established, including border quarantine for 14 days with polymerase chain reaction (PCR) tests, mandatory wearing of face masks in public areas, and forbidden large crowd gathering. Confirmed COVID-19 case number was reported daily by the Taiwan Centers for Disease Control (https://www.cdc.gov.tw/). All COVID-19 information was well documented and published by the government.

In Taiwan, SARS-CoV-2 vaccine has been available since June 2021. Some of the KTRs without COVID-19 history at the National Taiwan University Hospital (NTUH) were recruited in July 2021 for an observational vaccination effect study. After obtaining informed consent, blood samples were collected before (if available) and about 28 days after the first dose and 28, 90, and 180 days after the second dose. T and B cell responses after vaccination were analyzed as previously reported [4], which are briefly described in the next paragraph.

All KTRs over 18 years old undergoing regular follow-ups at NTUH outpatient clinic of the surgery department from April to August 2022, without confirmed COVID-19 before April 2022, were recruited in this retrospective study. Of these patients, in those with evidence of vaccination effect, vaccination dosage, clinical data, patient demographic profile, immunosuppressant usage, graft function, comorbidities, T and B cell responses (when available), and COVID-19 status were reviewed.

### Quantification of Immune Response After Vaccination

Spike protein-specific T cell response was determined by a SARS-CoV-2 interferon (IFN)-γ release assay (IGRA) kit (Quan-T-Cell SARS-CoV-2, Euroimmun Medizinische Labordiagnostica, Luebeck, Germany). The value of IGRA was considered a positive response if IFN-γ concentration was >100 (mIU/mL), according to the manufacturer’s instructions.

B cell response was determined by antibody concentration using an electrochemiluminescence immunoassay kit for spike and nucleocapsid protein (Elecsys Anti- SARS-CoV-2 S and Elecsys Anti- SARS-CoV-2, Roche) using a Cobas 411 analyzer. A value ≥ 0.8 U/mL was considered a positive response according to the manufacturer’s instructions. The Elecsys unit (U/mL) for antibody titer can be transformed into a binding antibody unit (BAU/mL) determined by the WHO using equation U = 0.972 × BAU.

### Data Analysis

Continuous variables are presented as the mean ± standard deviation for patients’ clinical profiles and compared using ordinary one-way ANOVA in three groups or more. The variables included age, transplant duration, serum tacrolimus level, serum creatinine, mycophenolate mofetil (MMF), and daily steroid doses. Student’s t-test was used for comparison of continuous variables and antibody and IGRA titers between two groups. Categorical variables, including sex, transplant type (cardaveric or living related transplantation), mTOR inhibitor usage, hypertension, diabetes mellitus, dyslipidemia, and hyperuricemia were analyzed using the chi-square test.

The standardized mortality ratio (SMR) was calculated for comparison between Taiwan’s general population and KTRs. Age-and-sex specific COVID-19 rate for the general population was obtained from the website of the Taiwan government, including the Taiwan National Development Council and the Ministry of Health (https://covid-19.nchc.org.tw/) and Welfare (https://www.cdc.gov.tw/).

We compared the cumulative incidence of COVID-19 and hospitalization between different groups of KTRs, defined by different vaccine dosage or antibody and IGRA levels, using the Kaplan-Meier test. The correlation between Ab and IGRA titer was determined by simple linear regression. The risk factors for COVID-19 and hospitalization were determined by simple logistic regression and further by Cox proportional hazards regression analysis.

A two-tailed test with *p* < 0.05 was considered statistically significant between groups. Statistical analysis was performed using GraphPad Prism 9.3.1 (GraphPad Software, LLC, CA, United States).

## Results

### Patient Demographic Data

**FIGURE 1:**
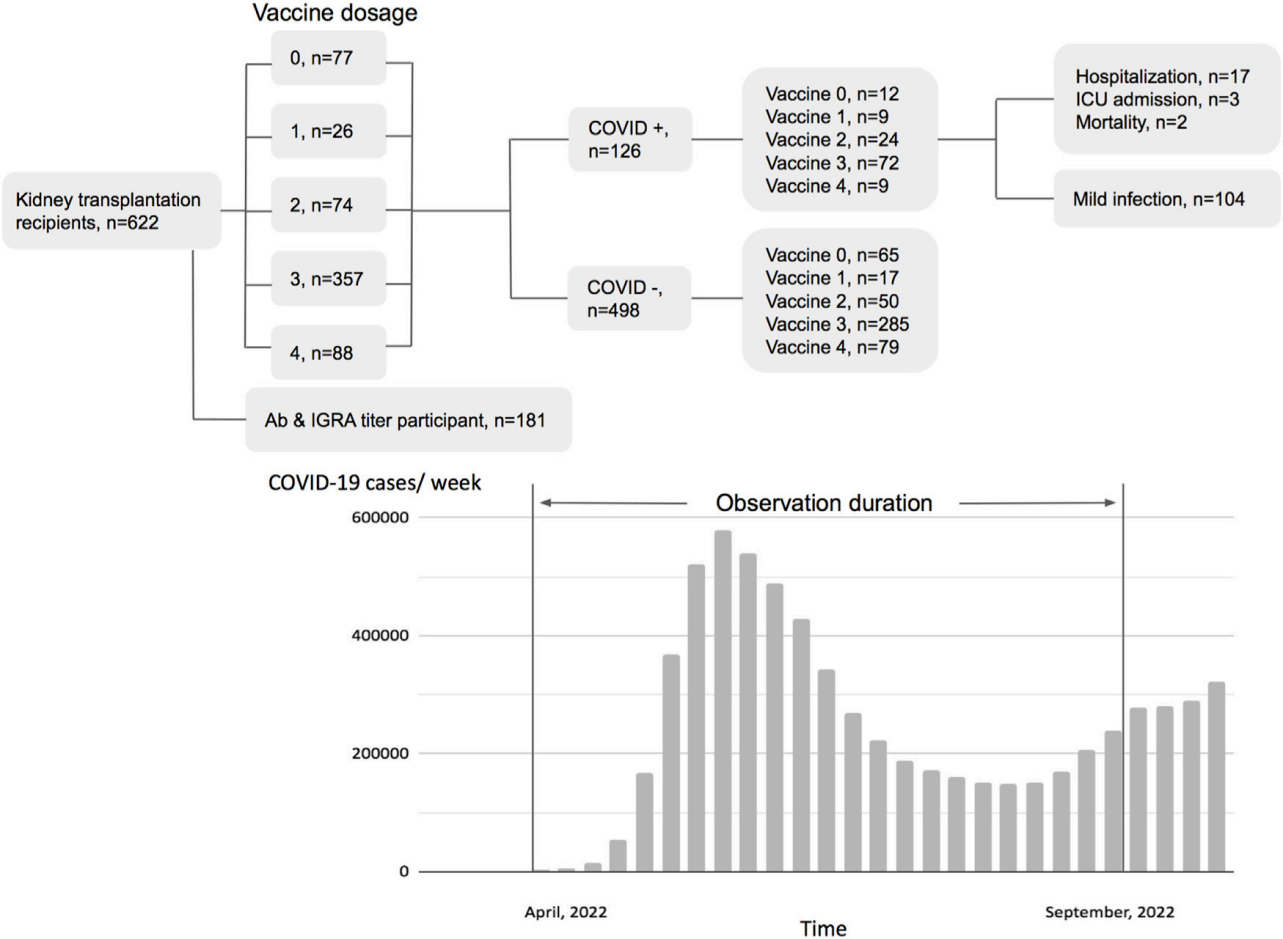
Flow chart of patient distribution by vaccination status and pandemic status of the general population in Taiwan.

**FIGURE 2:**
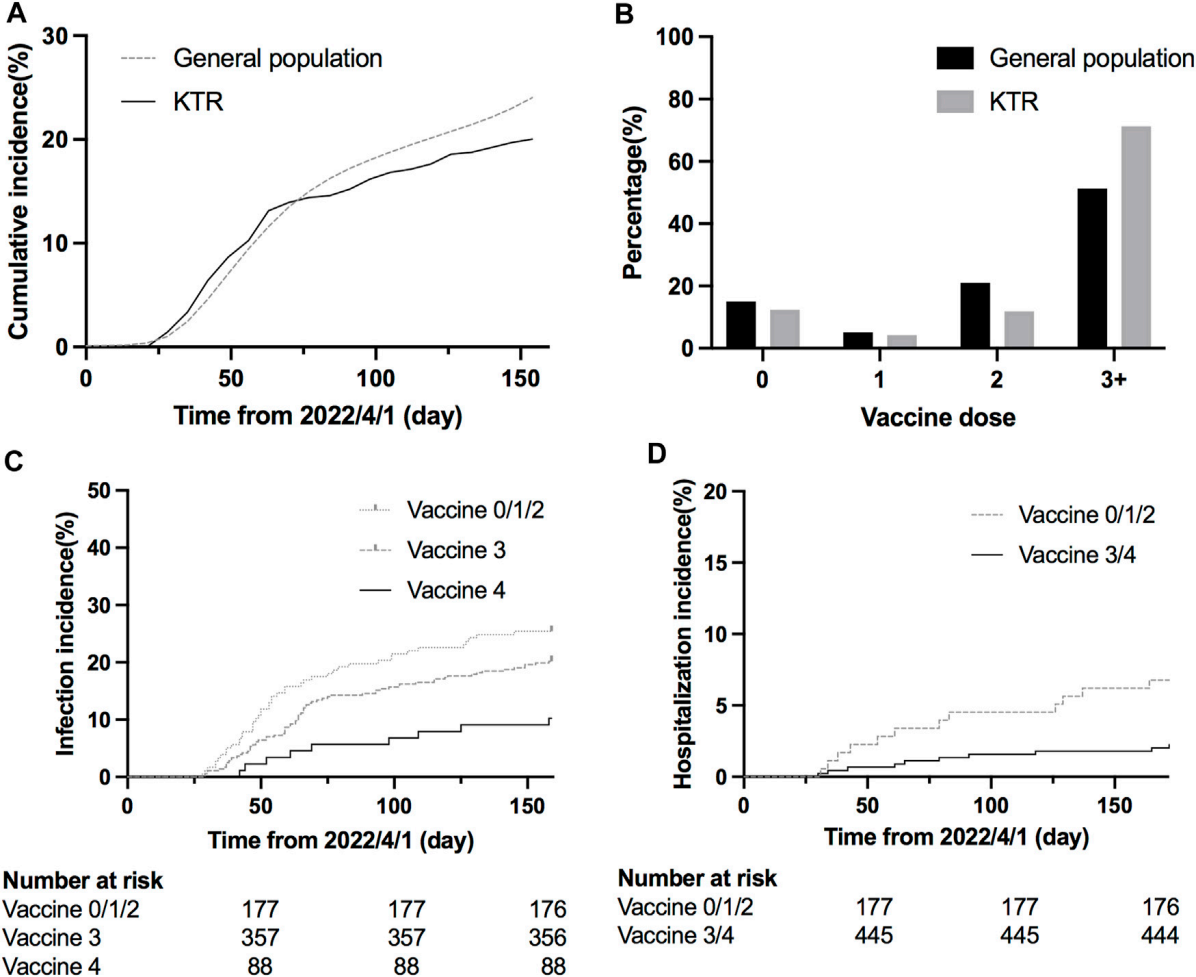
**(A)** Cumulative COVID-19 incidence since the COVID-19 outbreak (SMR = 0.80, 95% CI 0.66–0.95). **(B)** Percentage of KTRs and general population according to different vaccination doses before the outbreak **(C)** Cumulative COVID-19 incidence according to different doses (hazard ratio 0.48, 95% CI 0.28–0.81, *p* = 0.0289 for dose 4 vs. dose 3; hazard ratio 0.36, 95% CI 0.21–0.63, *p* = 0.0036 for dose 4 vs. dose 0/1/2; *p* = 0.1352 for dose 3 vs. dose 0/1/2). **(D)** Cumulative COVID-19 hospitalization incidence according to different doses (hazard ratio 0.32, 95% CI 0.13–0.82, *p* = 0.0055 for dose 3/4 vs. dose 0/1/2).

During April 2022 to August 2022, 622 KTRs were regularly attending the surgery department of the NTUH. One hundred twenty-six were diagnosed with COVID-19 by a home antigen test or PCR examination in the hospital (Figure 1). About 14% (22/126) of infected patients were hospitalized for medical treatment, and there were two mortality cases. Compared to the general population in Taiwan, the infection rate was lower (Standardized mortality ratio 0.80, 95% CI 0.66–0.95, Figure 2A), but the mortality rate seemed higher (1.6% vs. 0.18% for the general population) [18]. According to the vaccine doses, 77, 26, 74, 357, and 88 patients were vaccinated before the outbreak of COVID-19 with 0, 1, 2, 3, and 4 doses, respectively, which resembled the general population (Figure 2B) [18]. Based on vaccine type and dosage, there were 48 combinations for all KTRs and 24 combinations for KTRs receiving Ab and IGRA test. Among the various combinations, the two most common combinations were three doses of mRNA1273 (Moderna) (*n* = 128, 20.6%) and three doses of BNT162b2 (*n* = 51, 8.2%) (Supplementary Figures S1, S2). According to the Taiwan Centers for Disease Control (https://www.cdc.gov.tw/), although variants of the SARS-CoV-2 virus were only sampling tested, during April 1st to June 10th, Omicron BA.2 was the dominant variant (96%) in Taiwan. No BA.4 or BA.5 variant was detected until August 15th. However, the proportion of BA.4 and BA.5 variants increased rapidly to 5% and 40% respectively at the end of August. The patient characteristics regarding sex, age, transplant types, and immunosuppressants were are listed in Table 1 according to vaccine doses. For KTR with three doses, there were more male patients (53.8%), while KTR without vaccination had higher creatinine levels than of other groups.

**TABLE 1:** Patient characteristics.

	All	Vaccine 0	Vaccine 1	Vaccine 2	Vaccine 3	Vaccine 4	*p*-value
	*N* = 622	*n* = 77	*n* = 26	*n* = 74	*n* = 357	*n* = 88	
Male (%)	48.7	44.2	38.5	39.2	53.8	42.0	0.0467
Age (years)	53.61 ± 13.69	56.21 ± 14.18	50.85 ± 17.00	53.38 ± 14.46	52.83 ± 13.37	55.43 ± 12.68	0.1608
Living related transplant (%)	51.1	46.8	50.0	51.4	53.2	46.6	0.7228
Transplant duration in months	137.02 ± 101.6	155.37 ± 83.22	136.35 ± 84.81	131.89 ± 87.88	133.30 ± 110.91	140.17 ± 92.25	0.5227
Serum creatinine (mg/dL)	1.47 ± 0.99	1.66 ± 1.24	1.48 ± 0.87	1.49 ± 1.20	1.43 ± 0.88	1.42 ± 1.06	0.0002
Tacrolimus level (ng/mL) *N* (%)	4.24 ± 1.88	4.10 ± 1.63	3.71 ± 1.98	4.18 ± 2.10	4.29 ± 1.85	4.33 ± 2.00	0.5876
	(84.4%)	(77.9%)	(96.2%)	(86.5%)	(85.2%)	(81.8%)	
mTOR inhibitor (%)	59.2	49.4	57.7	55.4	61.3	63.6	0.3271
MMF daily dose (g) *N* (%)	0.93 ± 0.39	0.86 ± 0.46	0.75 ± 0.31	0.90 ± 0.40	0.94 ± 0.38	0.97 ± 0.38	0.1368
	(76.8%)	(72.7%)	(76.9%)	(74.3%)	(79.8%)	(70.5%)	
Hypertension (%)	61.4	62.3	61.5	52.7	63.3	60.2	0.5597
Diabetes (%)	22.0	20.8	15.4	13.5	24.1	23.9	0.2958
Dyslipidemia (%)	45.7	41.6	34.6	52.7	46.2	44.3	0.4951
Hyperuricemia (%)	39.9	36.4	38.5	37.8	42.0	36.4	0.7941

MMF, mycophenolate mofetil.

### Protection Effect According to Vaccine Doses

We retrospectively reviewed COVID-19 in KTR, caused mainly by Omicron BA.2, during the first wave of the outbreak from April to August 2022 [19, 20] (Figure 1), and compared the result with those of the general population according to the information published by Taiwan Centers for Disease Control. After the first wave, KTR with 4 doses had the lowest overall infection rate (10%) compared to 21/30/31% for vaccine doses 3/2/1, respectively (Supplementary Figure S3A). Meanwhile, for the risk analysis, KTR with 4 doses had significantly lower infection risk than those with other doses (Figure 2C, hazard ratio [HR] 0.48, 95% confidence interval [CI] 0.28–0.81, *p* = 0.0289 for dose 4 vs. dose 3; HR 0.36, 95% CI 0.21–0.63, *p* = 0.0036 for dose 4 vs. dose 0/1/2; HR 0.75, 95% CI 0.51–1.11, *p* = 0.1352 for dose 3 vs. dose 0/1/2). More than 3/4 of infected KTRs were isolated at home and had a smooth recovery. The number of hospitalized patients reduced in each group. We found that more than three doses of vaccine helped to reduce the overall hospitalization rate (Supplementary Figure S3B, Figure 2D, HR 0.32, 95% CI 0.13–0.82, *p* = 0.0055 for dose 3/4 vs. dose 0/1/2). Other conditions could confound the effect of vaccine dosage. We then performed Cox proportional hazards regression analysis, which showed that more than three doses (HR 0.59, 95% CI 0.40–0.88, *p* = 0.0084) and longer transplantation duration (HR 1.00, 95% CI 0.99–1.00, *p* = 0.0101) were the two protection factors (Table 2) for COVID infection. Besides, vaccination with more than three doses was the only protective factor against hospitalization (Table 3, HR 0.37, 95% CI 0.15–0.90, *p* = 0.0269).

**TABLE 2:** Factors associated with COVID-19 (*n* = 622).

Variable	Univariate analysis			Cox regression		
	Odds ratio (OR)	OR 95% CI	*p*-value	Hazard ratio (HR)	HR 95% CI	*p*-value
Female	0.83	0.56–1.22	0.3368	0.90	0.61–1.32	0.5894
Age	1.00	0.98–1.01	0.6338			
Vaccine ≥3 doses	0.65	0.43–0.99	0.0441	0.59	0.40–0.88	0.0084
Transplant duration	1.00	0.99–1.00	0.0002	1.00	0.99–1.00	0.0101
Creatinine level	1.03	0.84–1.24	0.7569			
Tacrolimus	1.14	1.03–1.27	0.0111	1.07	0.95–1.18	0.2431
mTOR inhibitor use	0.74	0.50–1.09	0.1284	0.85	0.56–1.30	0.4538
MMF	1.07	0.73–1.55	0.7439			
Hypertension	1.28	0.85–1.94	0.2403	1.14	0.76–1.72	0.5335
Diabetes	1.41	0.89–2.20	0.1309	1.52	0.99–2.29	0.0504

CI, confidence interval; MMF, mycophenolate mofetil.

**TABLE 3:** Factors associated with hospitalization due to COVID-19 (*n* = 622).

Variable	Univariate analysis			Cox regression		
	Odds ratio (OR)	OR 95% CI	*p*-value	Hazard ratio (HR)	HR 95% CI	*p*-value
Female	0.64	0.26–1.51	0.3177	0.63	0.25–1.54	0.3125
Age	1.00	0.97–1.03	0.7691			
Vaccine ≥3 doses	0.32	0.13–0.75	0.0085	0.37	0.15–0.90	0.0269
Transplant duration	1.00	1.00–1.00	0.9337			
Creatinine level	1.44	1.10–1.82	0.0034	1.23	0.91–1.56	0.1247
Tacrolimus	0.92	0.70–1.15	0.4987	0.89	0.67–1.13	0.3642
mTOR inhibitor use	0.47	0.19–1.10	0.0832	0.46	0.18–1.20	0.1154
MMF	1.19	0.53–2.73	0.6703			
Hypertension	2.93	1.08–10.24	0.0545	3.24	1.07–13.98	0.0632
Diabetes	1.05	0.34–2.70	0.9323			

CI, confidence interval; MMF, mycophenolate mofetil.

### Measurement of Immune Response

**FIGURE 3:**
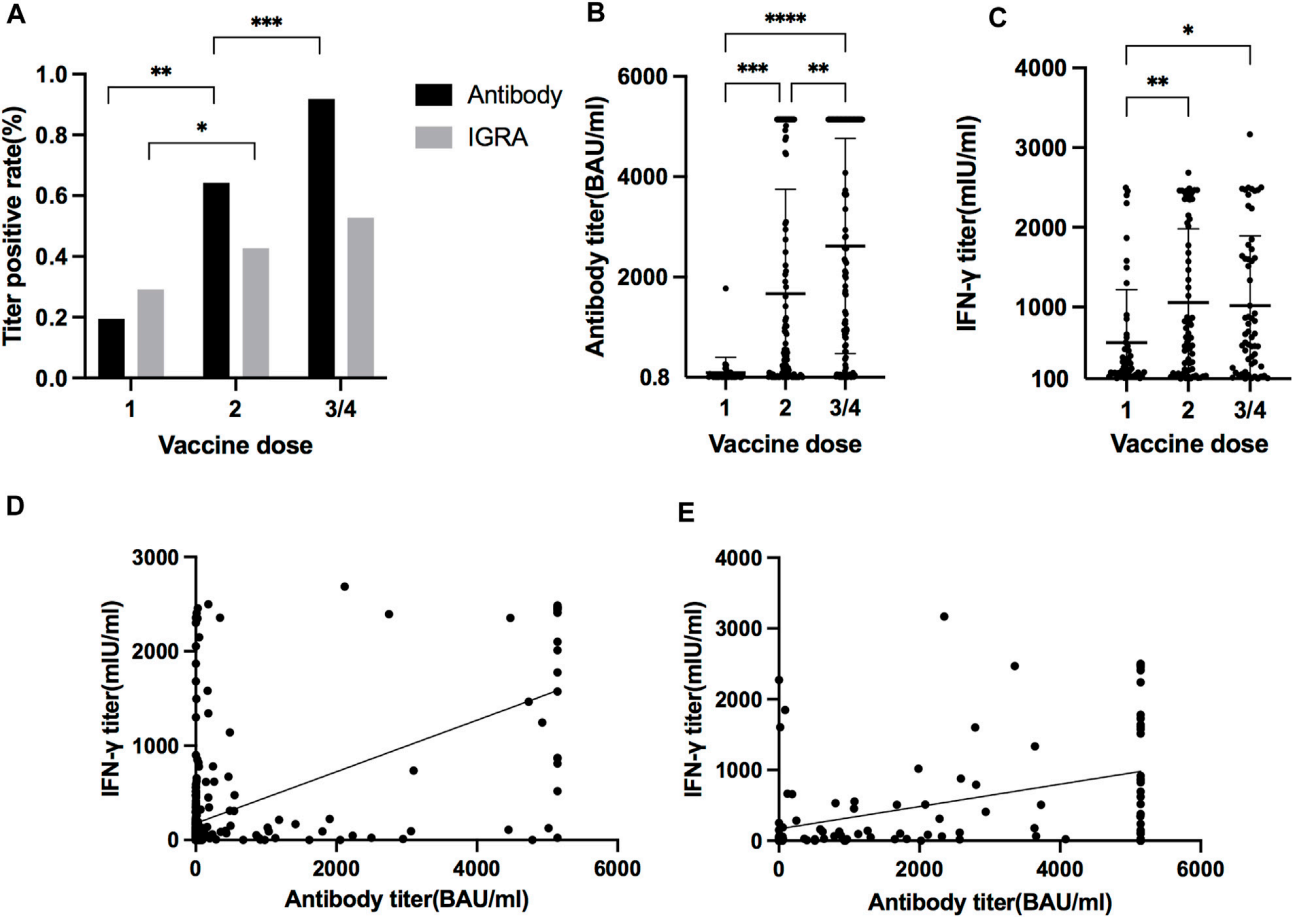
**(A)** Positive antibody and IGRA measurements rate after different vaccination doses. (*p = 0.0124, **p < 0.0001, ***p < 0.0001) **(B)** Antibody titer after different dosages of vaccination. (**p = 0.0017, ***p = 0.0002, ****p < 0.0001) **(C)** IFN-γ titer after different dosages of vaccination. (*p = 0.0113, **p = 0.0035) **(D)** Correlation between antibody titer and IFN-γ titer after first and second dose of vaccines (slope 0.27, 95% CI 0.23–0.32, *r* ^2^ 0.33, *p* < 0.0001). **(E)** Correlation between antibody titer and IFN-γ titer after third and fourth dose of vaccines (slope 0.16, 95% CI 0.096–0.22, *r* ^2^ 0.18, *p* < 0.0001).

Among the 622 KTRs, there were 181 KTRs undergoing antibody and IFN-γ assay after each dose of vaccine. Patient characteristics are presented in Table 4, and 112 KTRs (61.88%) received more than three doses. For antibody measurement, both the positive detection rate and titer increased with the doses (Figures 3A, B). For the IFN-γ assay, there was an increasing response after the second dose, but the trend became non-significant after the third dose, both in positive rate and IFN-γ titers (Figures 3A, C). The correlation between antibody and IFN-γ titer was more robust in the first two doses than in the last two doses (Figures 3D, E).

**TABLE 4:** Patient characteristics of KTRs with measurement of immune responses after vaccination.

Variables	Vaccine 0,1,2 *n* = 69	Vaccine 3,4 *n* = 112	*p*-value
Male (%)	44.9	41.1	0.6444
Age (year)	52.22 ± 12.95	54.93 ± 11.83	0.1505
Living related transplant (%)	43.5	42.0	0.6399
Transplant duration in months	132.77 ± 102.94	112.61 ± 84.19	0.1529
Serum creatinine (mg/dL)	1.43 ± 1.04	1.22 ± 0.43	0.0612
Tacrolimus level (ng/mL)	4.43 ± 1.29	4.79 ± 1.64	0.1802
mTOR inhibitor (%)	49.3	57.1	0.3573
MMF daily dose (g)	0.68 ± 0.53	0.66 ± 0.46	0.8567
Steroid daily dose (mg)	3.75 ± 3.03	3.42 ± 2.32	0.4107
Hypertension (%)	63.8	66.1	0.7510
Diabetes (%)	20.3	22.3	0.8529
Dyslipidemia (%)	44.9	50.9	0.4485
Hyperuricemia (%)	46.4	36.6	0.2142

KTR, kidney transplant recipient; MMF, mycophenolate mofetil.

### Infection Risk According to Immune Responses by Vaccination

**FIGURE 4:**
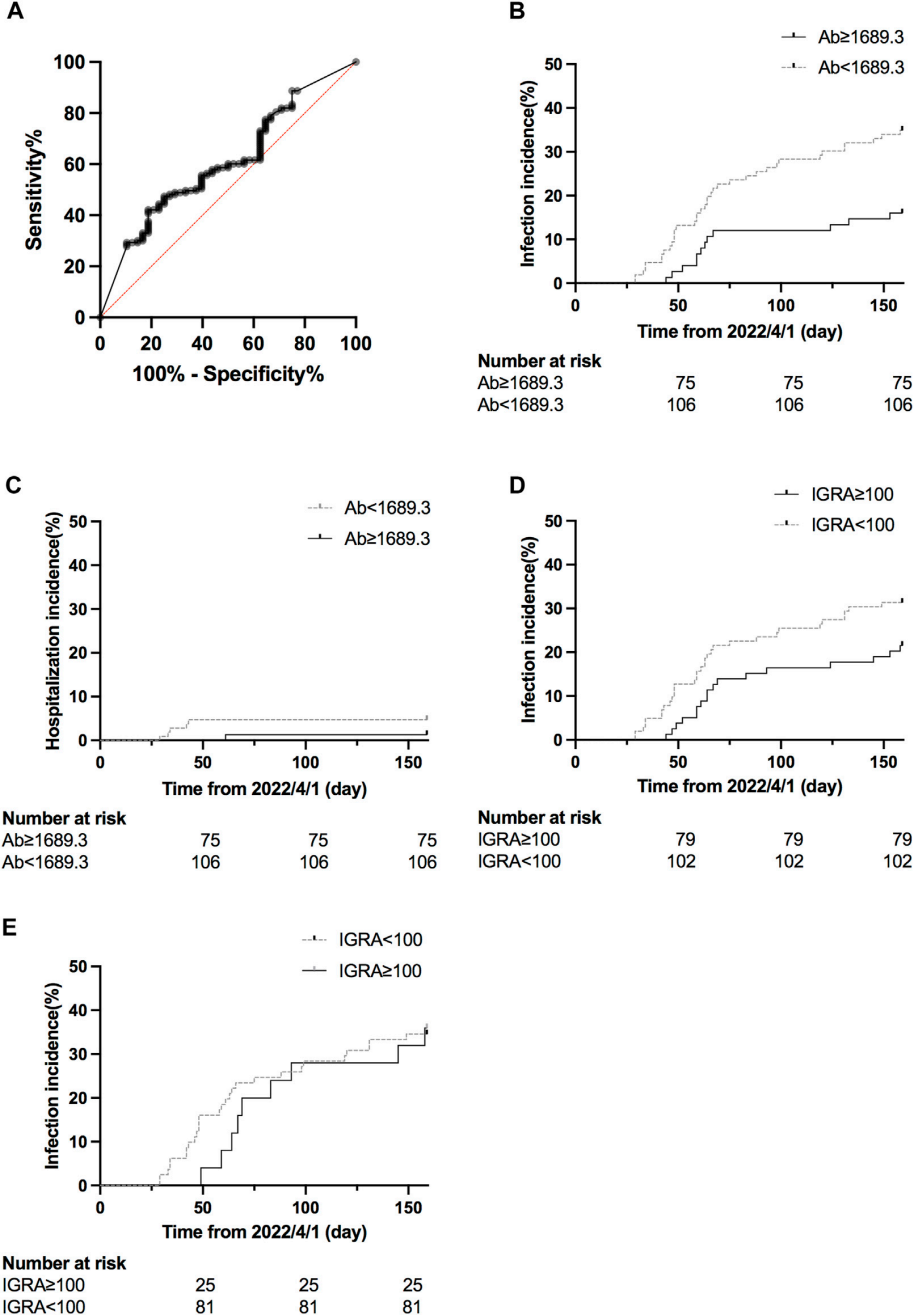
**(A)** ROC curve of infection status and antibody titer. The area under the curve was 0.61, 95% CI 0.52–0.61, *p* = 0.0271. **(B)** Cumulative COVID-19 incidence for antibody titer ≥1,689.3 BAU/mL vs. antibody titer <1,689.3 BAU/mL (hazard ratio 0.41, 95% CI 0.23–0.71, *p* = 0.0049). **(C)** Cumulative hospitalization incidence for antibody titer ≥1,689.3 BAU/mL vs antibody titer <1,689.3 BAU/mL (hazard ratio 0.28, 95% CI 0.05–1.40, *p* = 0.2083). **(D)** Cumulative COVID-19 incidence for IFN-γ titer ≥100 U/mL vs < 100 U/mL (hazard ratio 0.62, 95% CI 0.23–0.71, *p* = 0.1123). **(E)** Cumulative COVID-19 incidence for IFN-γ titer ≥100 U/mL vs <100 U/mL for patients with antibody titer <1,689.3 BAU/mL (hazard ratio 0.96, 95% CI 0.46–2.03, *p* = 0.9249).

It has been reported that higher antibody titer provided better protection against SARS-CoV-2 [21]. We performed a receiver characteristics curve (ROC) analysis to determine a cut-off value of 1642 U/mL (1,689.3 BAU/mL) (Figure 4A; Supplementary Table S2), and KTRs with a titer above this level had a significantly lower risk for infection (HR 0.41, 95% CI 0.23–0.71, *p* = 0.0049, Figure 4B) but not hospitalization (HR 0.28, 95% CI 0.05–1.40, *p* = 0.2083, Figure 4C). This might be due to low incidence in both groups (1/75 for titer ≥1,689.3 vs. 5/106 for titer <1,689.3). In contrast, a positive IGRA test did not show a significant protective effect for infection (HR 0.62, 95% CI 0.23–0.71, *p* = 0.1123, Figure 4D). We further analyzed the infection risk for KTR with antibody titers lower than 1,689.3 BAU/mL (*n* = 106) to verify if an antibody masked the effect of the cellular response. Nevertheless, there was no difference between KTRs with and without positive IGRA results (Figure 4E). To identify the influence of confounding factors, we then performed Cox regression analysis for risk of infection (Table 5) or hospitalization (Supplementary Table S1). Antibody titer >1,689.3 BAU/mL was the only significant factor (HR 0.46, 95% CI 0.21–0.95, *p* = 0.0412) against infection but not with >3 doses of vaccine (HR 0.52, 95% CI 0.27–1.11, *p* = 0.0714).

**TABLE 5:** Factors associated with COVID-19 for patients with measurement of immune response after vaccination (*n* = 181).

Variable	Univariate analysis			Cox regression		
	Odds ratio (OR)	OR 95% CI	*p*-value	Hazard ratio (HR)	HR 95% CI	*p*-value
Female	0.98	0.51–1.92	0.9583			
Age	0.98	0.95–1.01	0.117	1.00	0.97–1.02	0.8655
Vaccine ≥3 doses	0.35	0.14–0.86	0.0200	0.52	0.27–1.11	0.0714
Transplant duration	1.00	0.99–1.00	0.0334	1.00	0.99–1.00	0.2446
Creatinine level	1.03	0.61–1.59	0.8968			
Tacrolimus	1.03	0.82–1.28	0.8018			
mTOR inhibitor use	0.67	0.35–1.30	0.2372	0.82	0.46–1.47	0.5036
MMF	0.99	0.50–1.94	0.9733			
Steroid	1.15	1.02–1.32	0.0290	1.08	0.96–1.19	0.1605
Hypertension	0.89	0.45–1.79	0.7401			
Diabetes	1.47	0.67–3.13	0.3221	1.62	0.80–3.12	0.1635
Antibody titer ≥1,689.3 BAU/mL	0.36	0.16–0.72	0.0058	0.46	0.21–0.95	0.0412
Positive IGRA	0.60	0.30–1.17	0.1409	1.29	0.63–2.55	0.4647

CI, confidence interval; MMF, mycophenolate mofetil; BAU, binding antibody unit; IGRA, interferon-γ release assay.

## Discussion

Compared to previous studies conducted during the pandemic, this study demonstrated the effectiveness of multiple vaccine doses and antibody measurements before the outbreak of the Omicron variant, due to strict epidemic control in Taiwan. More than three doses of the vaccine provided significant protection for KTRs, and antibody titer of >1,689.3 BAU/mL may be a beneficial factor against SARS-CoV-2.

During the study period, vaccination was the only available method to prevent COVID-19, as monoclonal antibodies were not accessible at that time. Medications such as Ramdesivir, Paxlovid, and Monupiravir were only available for confirmed COVID-19 treatment in Taiwan. All the KTRs in this study received vaccines designed for the ancestral strain of SARS-CoV-2 as for the general population. Mean antibody titer against the spike protein increased with the sequential doses. Nevertheless, there was a tremendous interpatient variety. About 8% of KTRs still had no antibody response after more than three doses, which is different from that of the general population. Theoretically, KTRs should be more vulnerable to infection, but our result did not reveal this phenomenon, similar to the Danish report [10]. In Taiwan, home antigen tests and PCR tests in the hospital were officially recognized for SARS-CoV-2. Most people, including KTRs, had a test at home due to upper airway symptoms and then received medications by telemedicine from numerous local clinics and hospitals, a system established right after the outbreak. Under the same diagnostic criteria, for a short period, we believe that the infection rate reflected the real-world status. Hence, one of the possible explanations is that KTRs may take more protective measures, but still had similar results as others. Besides, multiple mutations in the spike protein resulted in antibody evasion and higher transmission ability by the Omicron variant [22, 23], which may further attenuate the different vaccine effects between KTR and the general population.

Meanwhile, more vaccine doses still showed protection effects in this study. There was a lower risk for infection and hospitalization for KTRs with three or more doses, especially four. Regarding mortality, there were only two cases in our cohort, making it difficult to draw a conclusion. Of the two patients who died, one had received two doses of vaccination, and the other had received three doses. Neither of them had antibody measurements, so vaccine effectiveness could not be confirmed. The literature has shown that antibodies evoked by the first-generation vaccine still affected the Omicron variant [24]. A higher titer is needed [12], which could be achieved by booster strategy. For KTRs, a meta-analysis showed a positive antibody detection rate of around 60% after the third dose, and the antibody titer also increased [1]. KTRs take various immunosuppressants that impede lymphocyte activation for antibody generation.

Moreover, the waning rate of antibodies is prominent in KTRs, even after a third dose [25]. Measurement of titer may help to identify KTRs with different risks and administer boosters to those with poor response to vaccination. For those already having a high antibody titer, the risk of side effects [26] for a booster may outweigh the limited benefit [27]. It should be noted that a high antibody titer does not equal a safe status. Our study shows that among patients with SARS-CoV-2, 49 patients had a known antibody titer, and six needed admission for further management. Most hospitalized patients (5/6) had antibodies <1,689.3 BAU/mL, but one patient had an antibody titer higher than 3,000 U/mL. For SOTR, that high antibody titer after booster did not represent equivalent neutralization capacity for the Omicron variant [28]. Hence, the result of antibody measurement should be interpreted with caution. However, it still has a more significant role in KRTs than in the general population for the risk stratification to decide between boosters. In this study, we also examined the result of IGRA as a cellular response to vaccination. Compared to antibody titer, IFN-γ level of cellular response assay did not increase significantly with the boosters after the third dose both based on the percentage of positive results and IFN-γ titers. All the KTRs were on immunosuppressants targeting mainly the T cells, hence, the response was suppressed [29]. In addition, it has been reported in the immunocompetent general population [30, 31] that T cell response could not be augmented by repeated boosters despite detectable SARS-CoV-2 specific T cell population after initial doses. Unlike virus infection, vaccination with booster doses did not provoke equivalent IFN-γ and IL-10 expression memory T cells. It is postulated that viral infections on the pulmonary site persist longer and stronger than intramuscular vaccination; they induce a robust inflammatory cytokine release, which enhances a more durable T-lymphocyte response [32] and generates more tissue-resident memory T cells [33]. In addition, we used circulating lymphocytes for the IFN-γ release assay, and the result may not reflect the response of local memory T cells evoked by boosters.

Although T cell response correlates positively to antibody response (Figures 3D, E), we did not find the effect of a positive IFN-γ response to SARS-CoV-2 in the prevention of infection, as reported in dialysis patients [34]. It has been shown that T cell response is crucial when humoral immunity is impaired [35]. Nevertheless, under strong antibody response, the possible secondary role of T cells could be masked [36]. Our subgroup analysis for KTRs with low or absent antibody titer did not show a protective effect against infection by a positive cellular response. We speculated that T cell response might be slower than antibody response, which could neutralize the virus at the first encounter. The cellular response might be more important for disease severity, which this small study with low admission requirements and rare mortality could not reveal.

There were several limitations in this study. First, under a pandemic status with limited availability of vaccines, KTRs received vaccines based on different platforms. We had previously shown that KTRs had weaker responses to all types of vaccines compared to the responses in general population, and the immunogenicity varied among the vaccine platforms [4]. In Taiwan, most people, including KTRs, received homologous vaccines for the first two doses. They could choose either an mRNA or protein subunit vaccine for the third dose as a personal preference. It is difficult to identify the effect of different vaccines, but we found that most (approximately 90%) KTRs would have detectable antibody titers after the fourth dose. The effect of multiple boosters was robust regardless of vaccine type. Second, we retrospectively reviewed the infection risk during an outbreak caused mainly by the Omicron strain BA.2 [19, 20], which might not represent a general protective effect against other strains. It was well known that the Omicron strain had more immune evasion than previous strains of the first generation vaccines. This study still showed a significant effect of vaccine doses, and further observation on different variants is needed. Third, our study did not perform an antibody neutralization test, and it is difficult to correlate directly between the protection effect and antibody measurement by anti-spike protein assay. We admitted the importance of the neuralization test according to different virus strains. However, the equipment and expense requirements may become a limitation in many medical institutions. Developing new economic tests for different virus variants might be necessary for more precise measurement.

In conclusion, this study showed a protective effect against SARS-CoV-2 according to vaccine doses and laboratory measurements in KTRs. Despite impaired immune function, KTR still had increasing responses after repeated vaccination. After the third dose, the protection effect became prominent but varied among patients. Measurement of antibodies could be helpful to determine individual risk and the need for further boosters. These findings provide evidence for a specific vaccination strategy in KTRs, who require more boosters than the general population to achieve an adequate antibody titer that may be necessary in a pandemic.

## Data Availability

The raw data supporting the conclusion of this article will be made available by the authors, without undue reservation.

## Supplementary Material

Supplementary Figure S1 All vaccine combinations received by all KTRs (n = 622) with the number and percentage of COVID-19 cases of each group.

Supplementary Figure S2 All vaccine combinations received by KTRs with antibody and IGRA titer (n = 181) with the number and percentage of COVID-19 cases of each group.

Supplementary Figure S3
**(A)** Comparison of COVID-19 rate between different vaccine doses. 31%, 30%, 21%, 10% for vaccine doses 1/2/3/4. * *p* = 0.0220, ** *p* = 0.0023, *** *p* = 0.0235 **(B)** Comparison of hospitalization rate between groups of different vaccine doses. 8%, 11%, 3%, 1% for vaccine doses 1/2/3/4. * *p* = 0.0035, ** *p* = 0.0119.

## Abbreviations

BAU, binding antibody unit; COVID-19, coronavirus disease 2019; IGRA, interferon-γ release assay; IFN-γ, interferon-γ; KTR, kidney transplant recipient; MMF, mycophenolate mofetil; SARS-CoV-2, severe acute respiratory syndrome coronavirus 2; SMR, Standardized mortality ratio; SOTR, Solid organ transplant recipient.
