# Supplementary figures and images for: Protective Effect of Vaccine Doses and Antibody Titers Against SARS-CoV-2 Infection in Kidney Transplant Recipients
Source: Transpl Int. 2023 Jun 13;36:11196. doi: 10.3389/ti.2023.11196 (PMC10294008; doi:10.3389/ti.2023.11196)

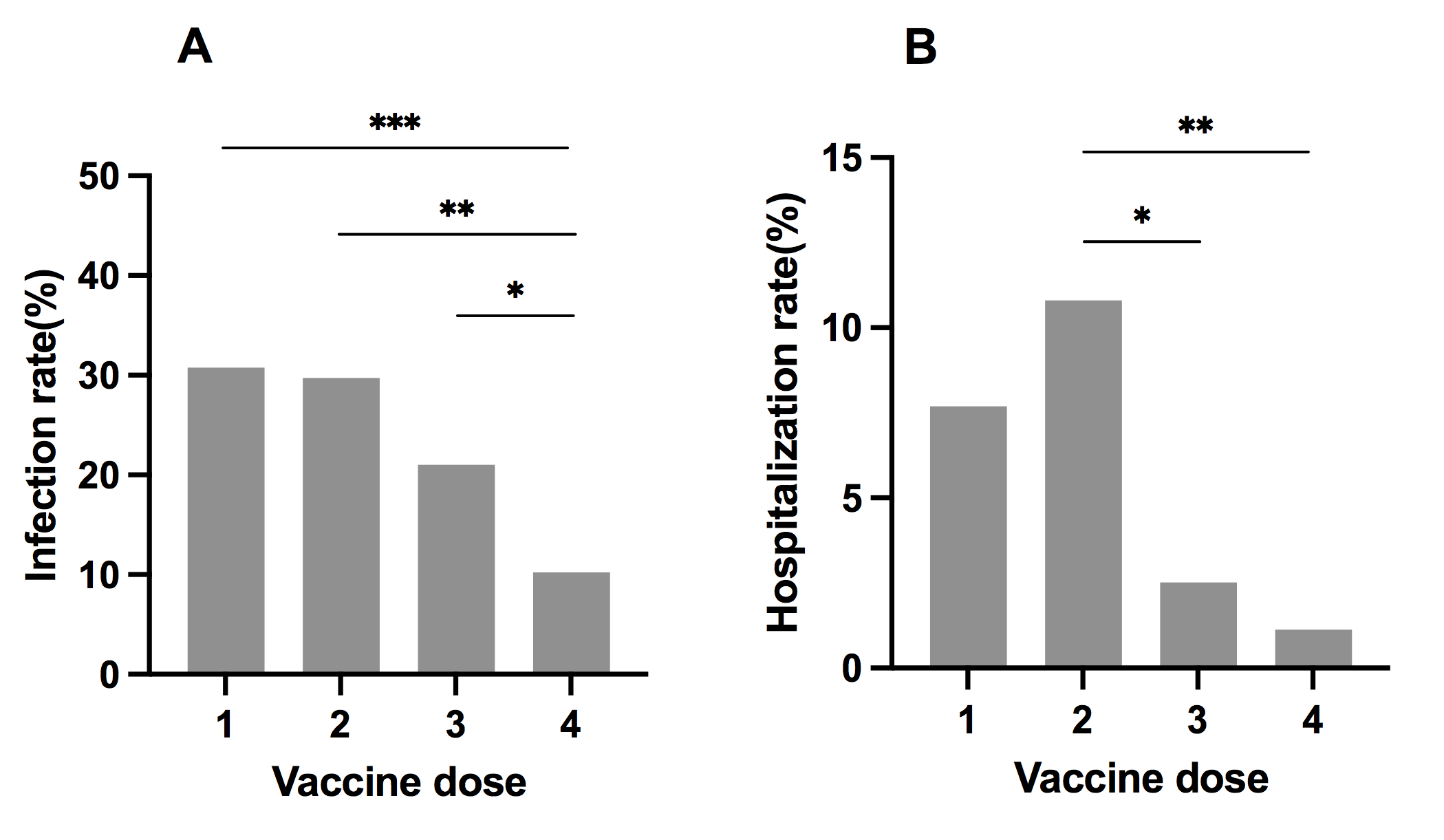

Supplement: Supplementary file 1 [file Image3.TIFF]

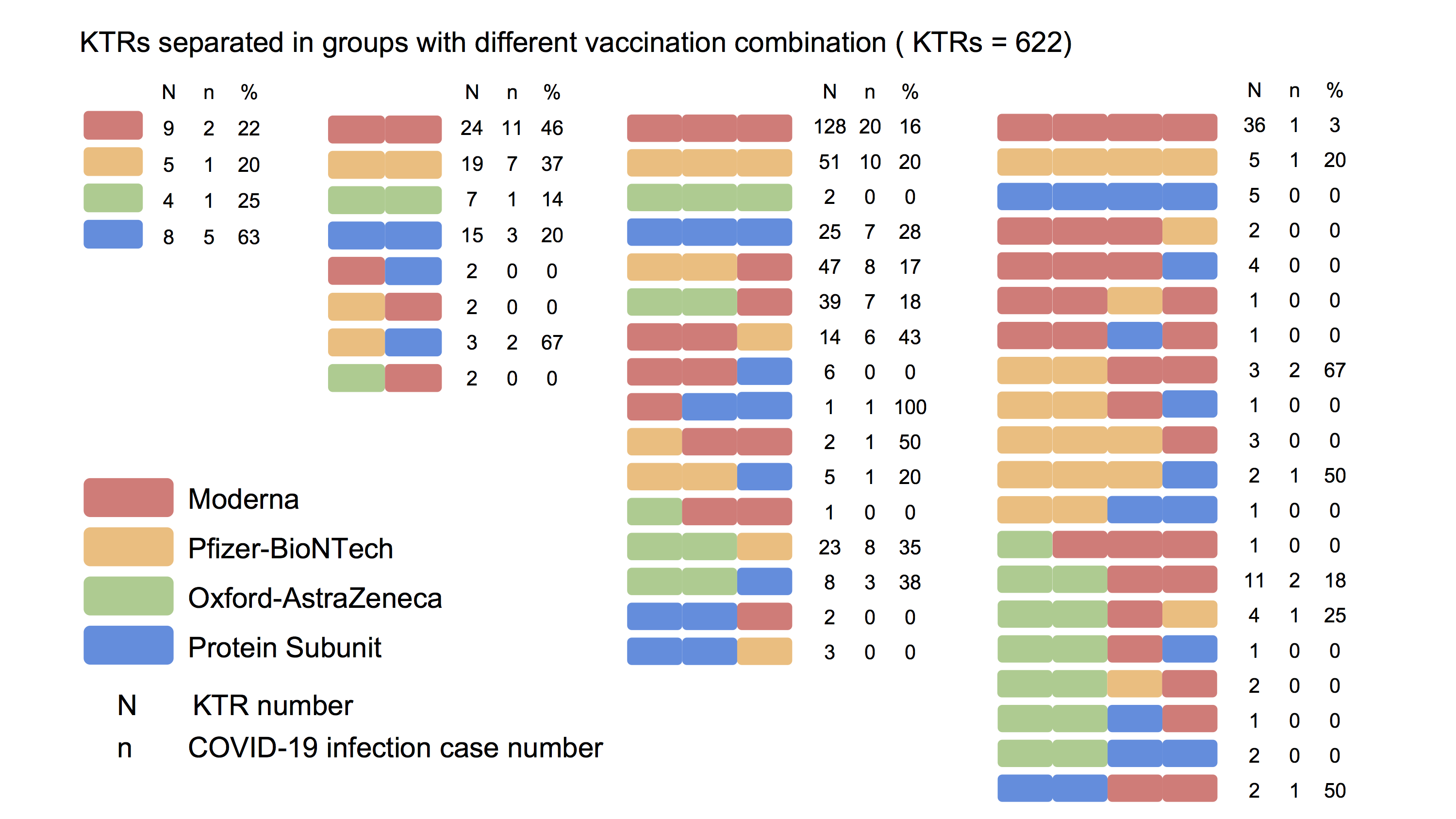

Supplement: Supplementary file 2 [file Image1.TIFF]

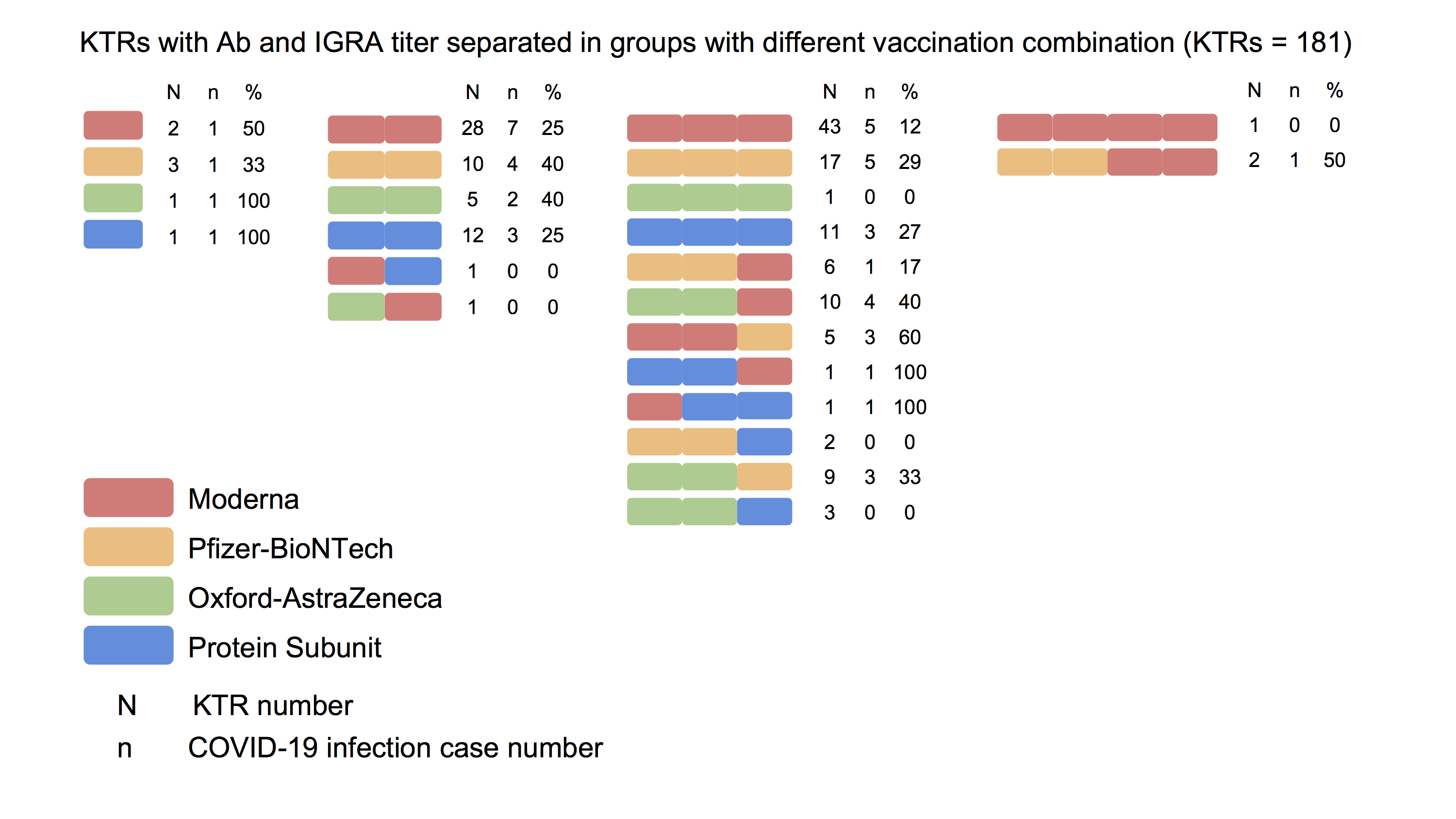

Supplement: Supplementary file 3 [file Image2.TIFF]
