# Supplementary material for: Protective Effect of Vaccine Doses and Antibody Titers Against SARS-CoV-2 Infection in Kidney Transplant Recipients
Source: Transpl Int. 2023 Jun 13;36:11196. doi: 10.3389/ti.2023.11196 (PMC10294008; doi:10.3389/ti.2023.11196)
Supplement: Supplementary file 4 [file DataSheet1.docx]

| **Supplementary Table 1**. Factors associated with hospitalization due to COVID-19 for patients with immune response measurements after vaccination (n=181). | | | | | | |
| --- | --- | --- | --- | --- | --- | --- |
|  | Univariate analysis | | | Cox regression | | |
| variable | Odds ratio (OR) | OR 95% CI | p value | Hazard ratio (HR) | HR 95% CI | p-value |
| Female | 0.14 | 0.01-0.89 | 0.0753 | 0.21 | 0.01-1.63 | 0.1876 |
| Age | 1.01 | 0.95-1.10 | 0.7196 |  |  |  |
| Vaccine ≥ 3 doses | 0.27 | 0.05-2.05 | 0.1474 | 0.19 | 0.02-1.70 | 0.1111 |
| Transplant duration | 0.99 | 0.98-1.00 | 0.3683 | 1.00 | 0.98-1.01 | 0.9446 |
| Creatinine level | 0.65 | 0.06-1.81 | 0.6623 |  |  |  |
| Tacrolimus | 1.01 | 0.59-1.67 | 0.9839 |  |  |  |
| mTOR inhibitor use | 0.41 | 0.06-2.16 | 0.3125 | 0.38 | 0.04-2.34 | 0.3273 |
| MMF | 5.27 | 0.95-37.31 | 0.0699 | 3.86 | 0.67-24.62 | 0.1309 |
| Steroid | 1.09 | 0.80-1.43 | 0.5637 | 1.01 | 0.61-1.48 | 0.9810 |
| Hypertension | 2.74 | 0.43-53.15 | 0.3619 | 1.54 | 0.16-34.30 | 0.7303 |
| Diabetes | 1.87 | 0.25-9.94 | 0.4816 | 1.30 | 0.13-8.94 | 0.7975 |
| Antibody titer ≥ 1689.3 BAU/mL | 0.27 | 0.01-1.74 | 0.2405 | 0.82 | 0.03-10.62 | 0.8838 |
| Positive IGRA | 0.25 | 0.01-1.58 | 0.2083 | 0.37 | 0.01-3.50 | 0.4514 |

CI, confidence interval; MMF, mycophenolate mofetil; IGRA, interferon-γ release assay

| **Supplementary Table 2**. ROC curve of infection status and antibody titer. Sensitivity, specificity, and likelihood ratio of the cutoff values of antibody titer between 1000 and 2000 U/mL. | | | | | |
| --- | --- | --- | --- | --- | --- |
|  | Sensitivity% | 95% CI (%) | Specificity | 95% CI (%) | Likelihood ratio |
| >1040 | 48.87 | 40.53-57.28 | 68.75 | 54.67-80.05 | 1.564 |
| >1072 | 48.87 | 40.53-57.28 | 70.83 | 56.82-81.76 | 1.676 |
| >1162 | 48.12 | 39.80-56.54 | 70.83 | 56.82-81.76 | 1.65 |
| >1270 | 48.12 | 39.80-56.54 | 72.92 | 59.00-83.43 | 1.777 |
| >1460 | 47.37 | 39.08-55.81 | 72.92 | 59.00-83.43 | 1.749 |
| >1642 | 47.37 | 39.08-55.81 | 75 | 61.22-85.08 | 1.895 |
| >1722 | 46.62 | 38.35-55.07 | 75 | 61.22-85.08 | 1.865 |
| >1828 | 45.86 | 37.63-54.33 | 75 | 61.22-85.08 | 1.835 |
| >1892 | 45.11 | 36.91-53.59 | 75 | 61.22-85.08 | 1.805 |
| >1912 | 44.36 | 36.19-52.84 | 75 | 61.22-85.08 | 1.774 |

CI, confidence interval
